# Supplementary figures and images for: A non-canonical Puf3p-binding sequence regulates CAT5/COQ7 mRNA under both fermentable and respiratory conditions in budding yeast
Source: PLoS One. 2023 Dec 15;18(12):e0295659. doi: 10.1371/journal.pone.0295659 (PMC10723686; doi:10.1371/journal.pone.0295659)

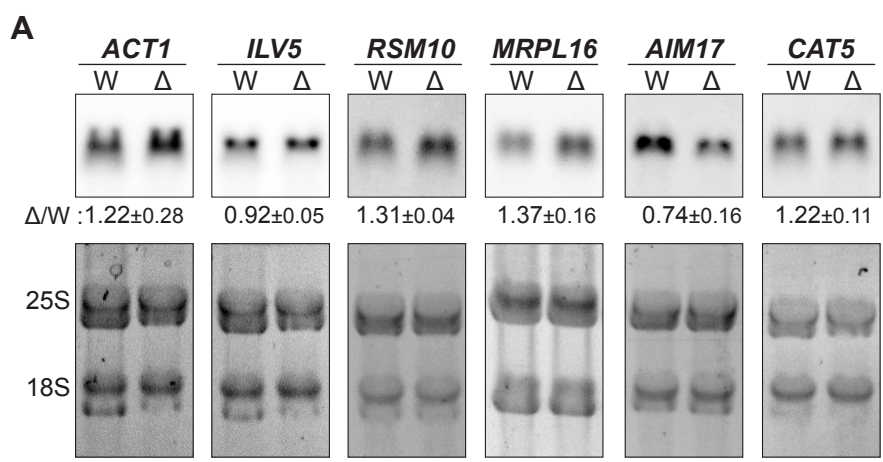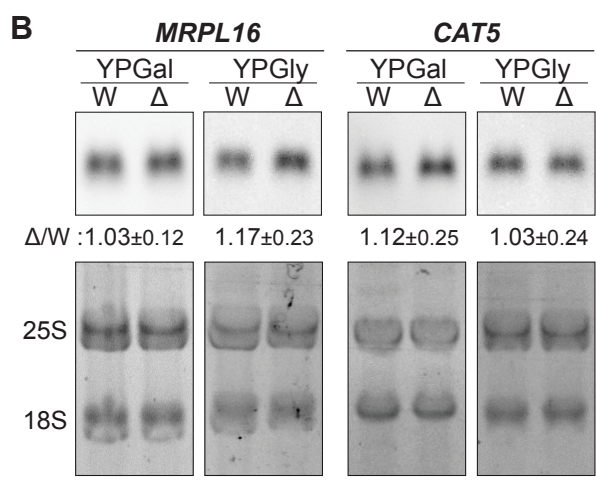

Supplement: S1 Fig — (A) Northern blot analysis of several monosome-enriched mRNAs. Total RNAs were isolated from the wild-type (W) and puf3Δ (Δ) yeasts grown in the fermentable medium (YPD), and those were subjected to the northern blotting for the indicated mRNA species. The numbers represent relative expression changes of mRNA abundance. The means and standard deviations of the mRNA signals (Δ/W, puf3Δ/WT) were calculated from n ≥ 3. Lower panels represent the total RNAs in each corresponding sample, visualized by GelRed staining prior to northern blotting. (B) Northern blot analysis of the MRPL16 and CAT5 mRNAs extracted from yeasts grown in respiratory media (YPGal and YPGly). The relative expression changes of the mRNAs were analyzed as described above. Lower panels represent the total RNAs in each corresponding sample, visualized by GelRed staining prior to northern blotting. (PDF) [file pone.0295659.s001.pdf]

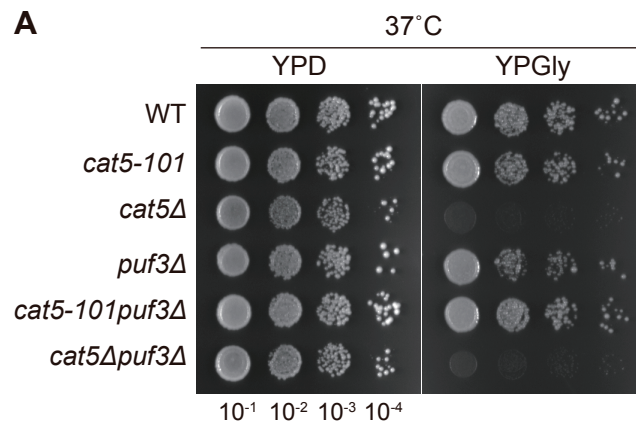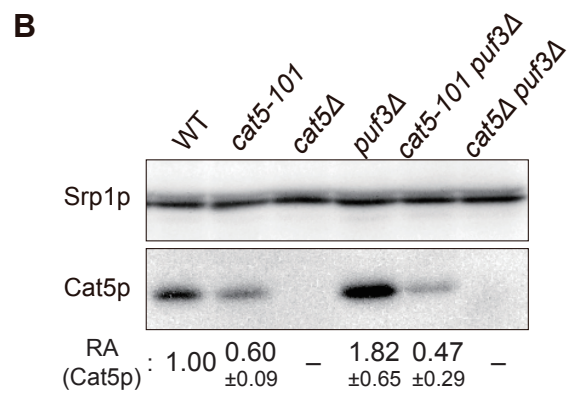

Supplement: S2 Fig — (A) Growth comparison among wild-type, cat5-101, and cat5Δ strains in the presence or absence of the PUF3 gene. Saturated cultures of the indicated strains were serially diluted by 10-fold as shown in the bottom and dropped onto YPD or YPGly plates, and incubated at 37°C. (B) Western blot analysis of Cat5p. The yeast strains in (A) were grown at 37°C in YPD, and subjected to western blotting. Srp1p was used as a loading control. The numbers under the gel images represent the mean ± standard deviation of the relative amount (RA) of Cat5p, quantified from n ≥ 3. The WT Cat5p expression was set to 1.00. (PDF) [file pone.0295659.s002.pdf]

Fig. 1A

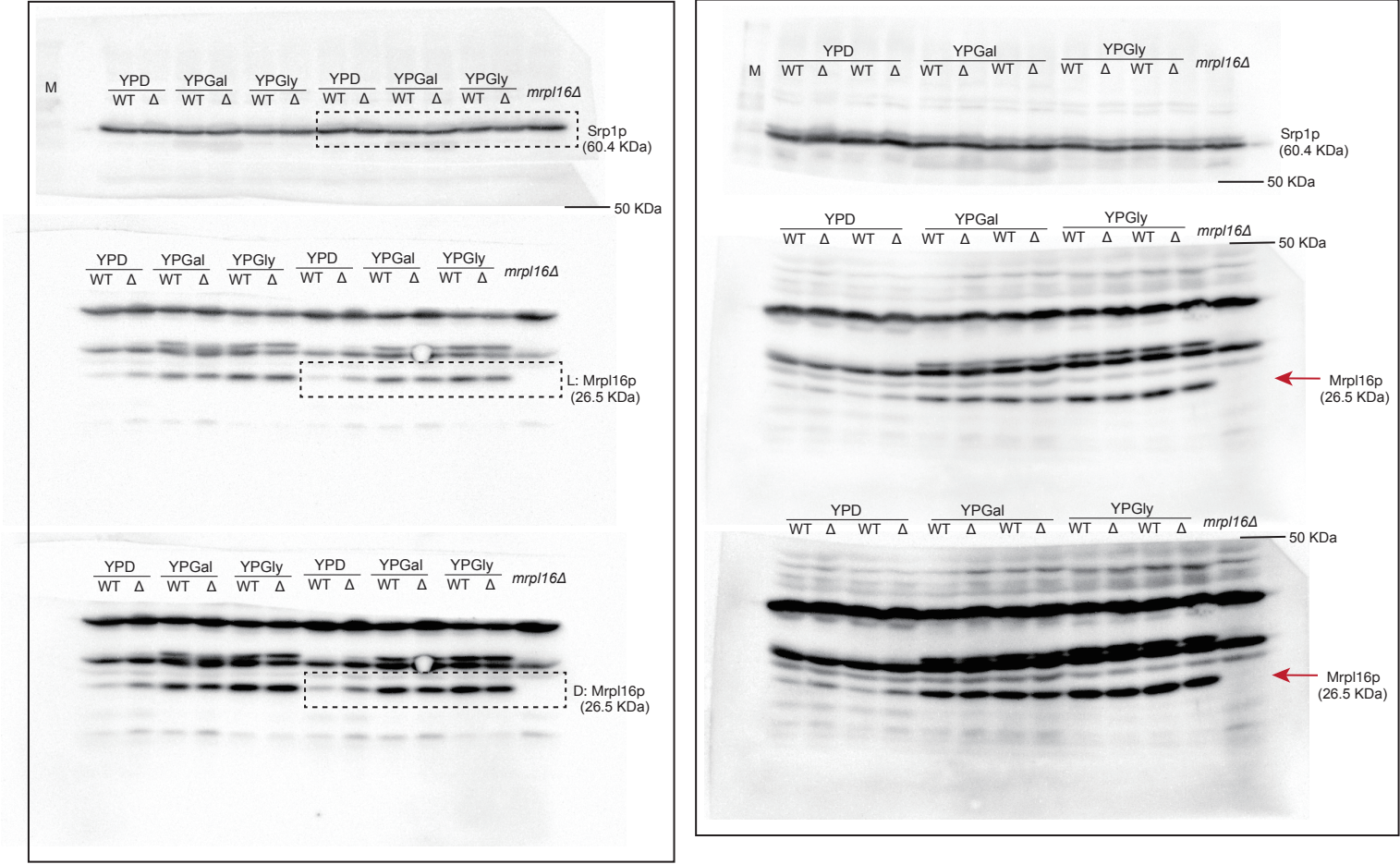

Fig. 1D

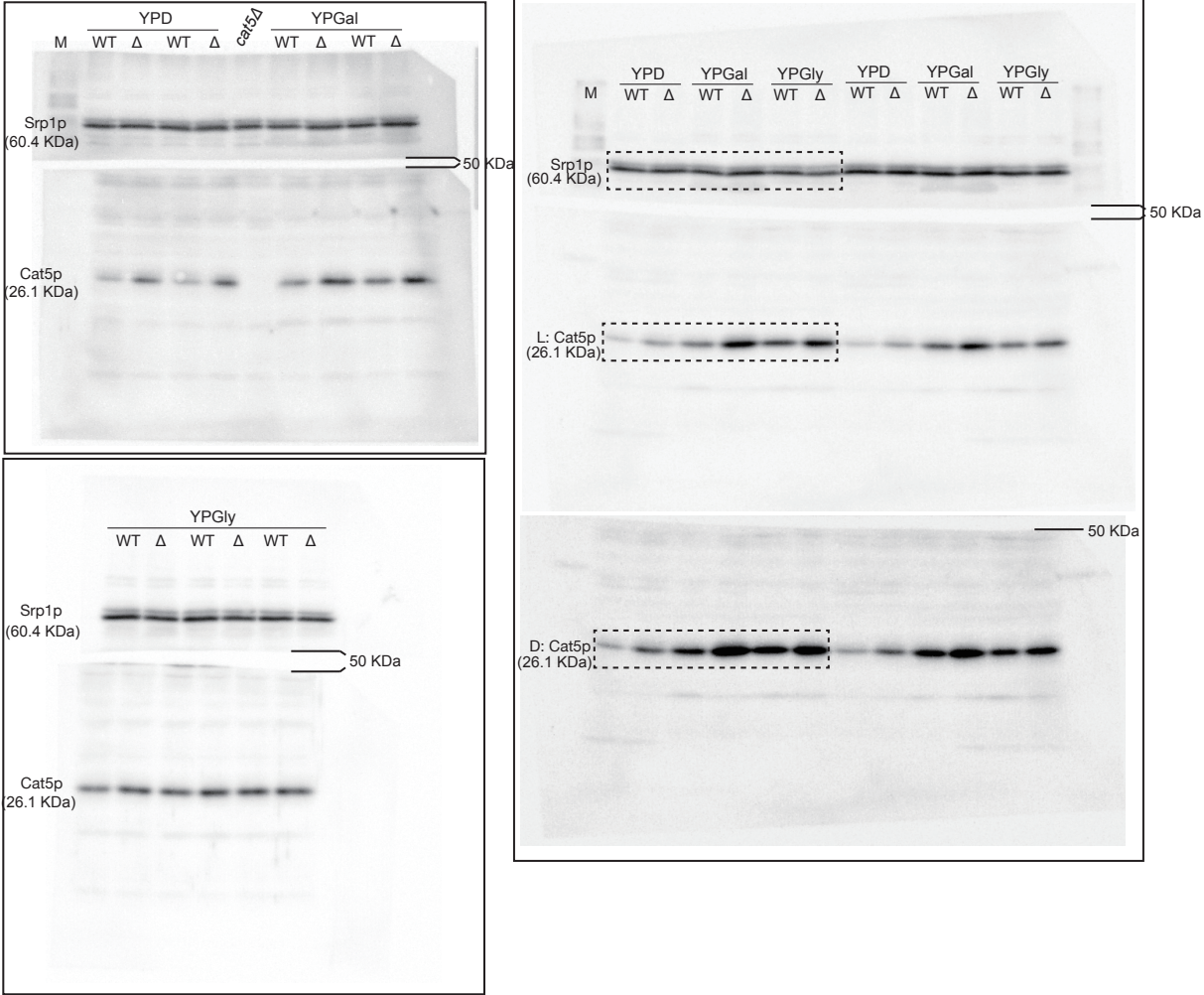

Supplement: S1 Raw images — (PDF) [file pone.0295659.s006.pdf]

Fig. 2D

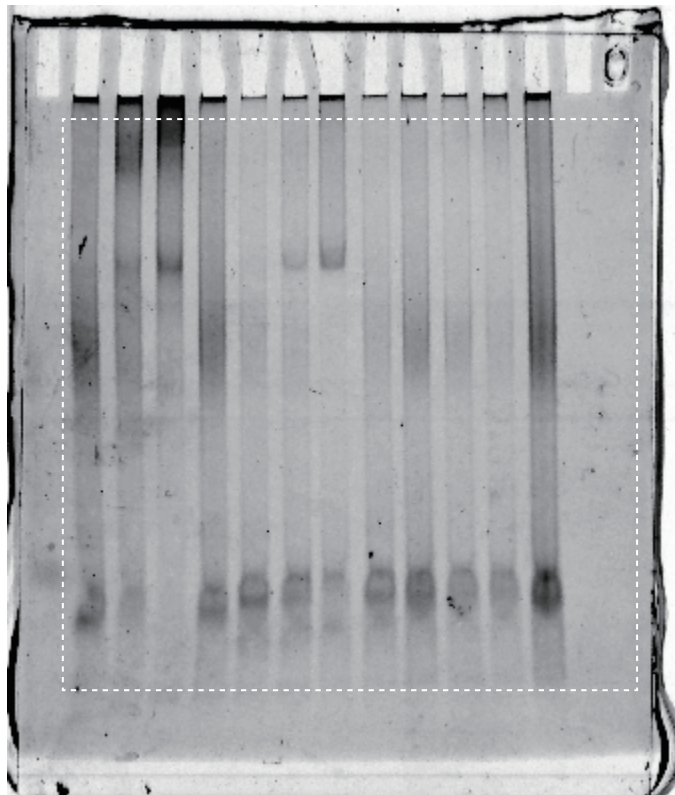

Fig. 2E

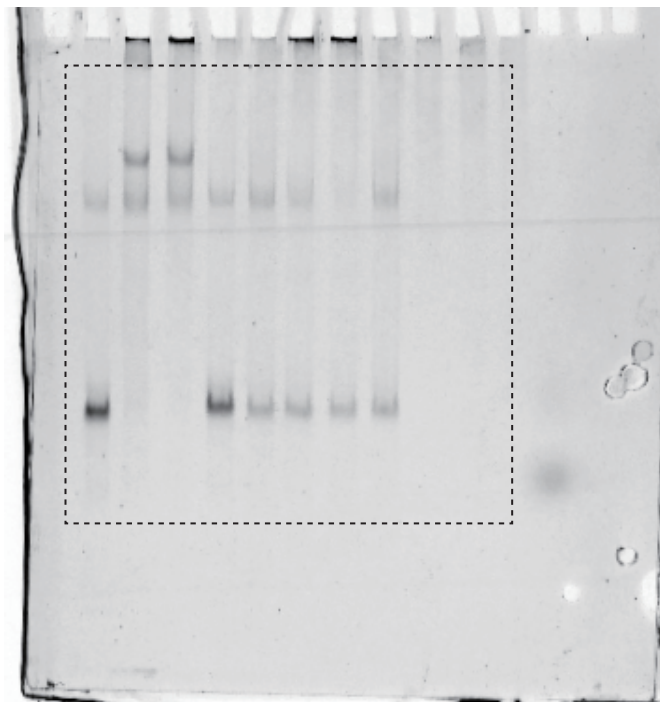

Supplement: S2 Raw images — (PDF) [file pone.0295659.s007.pdf]

Fig. 3B (30°C) and S2 Fig. B (37°C)

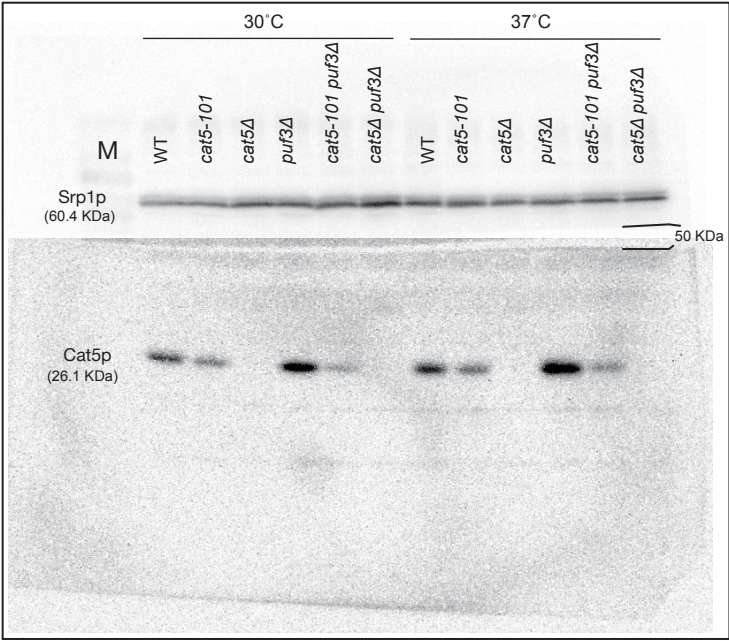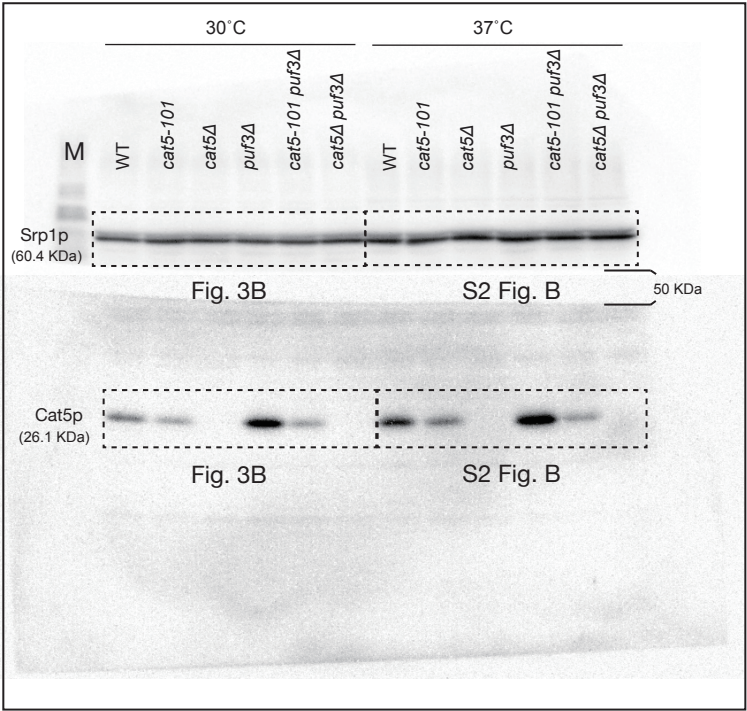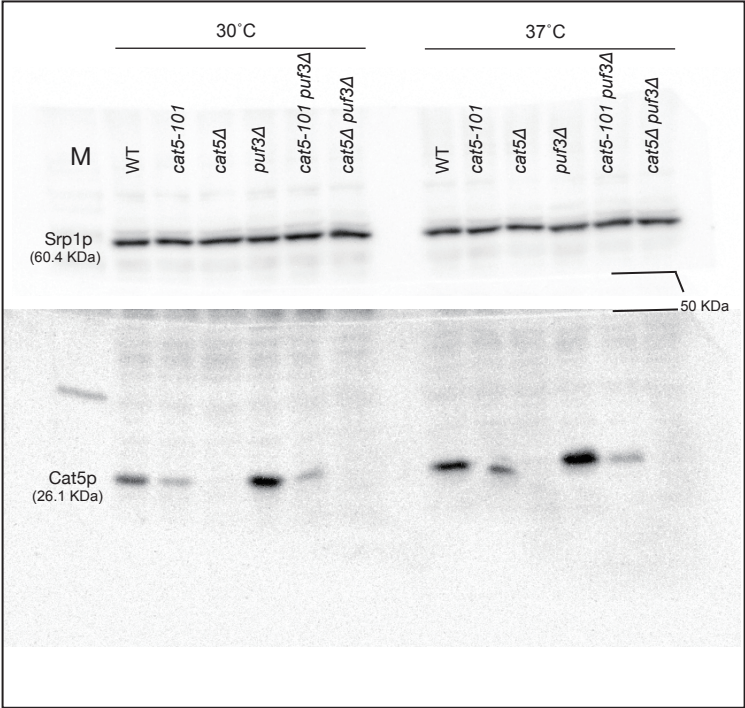

Fig. 3C

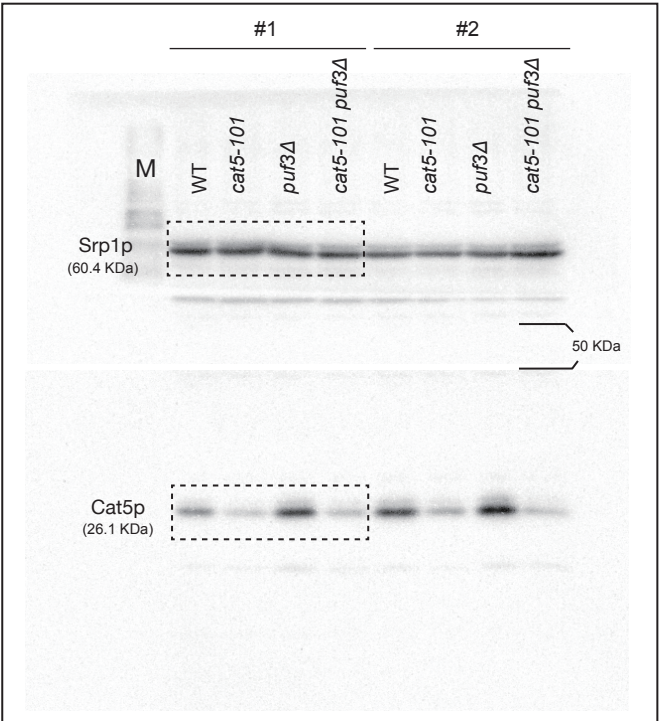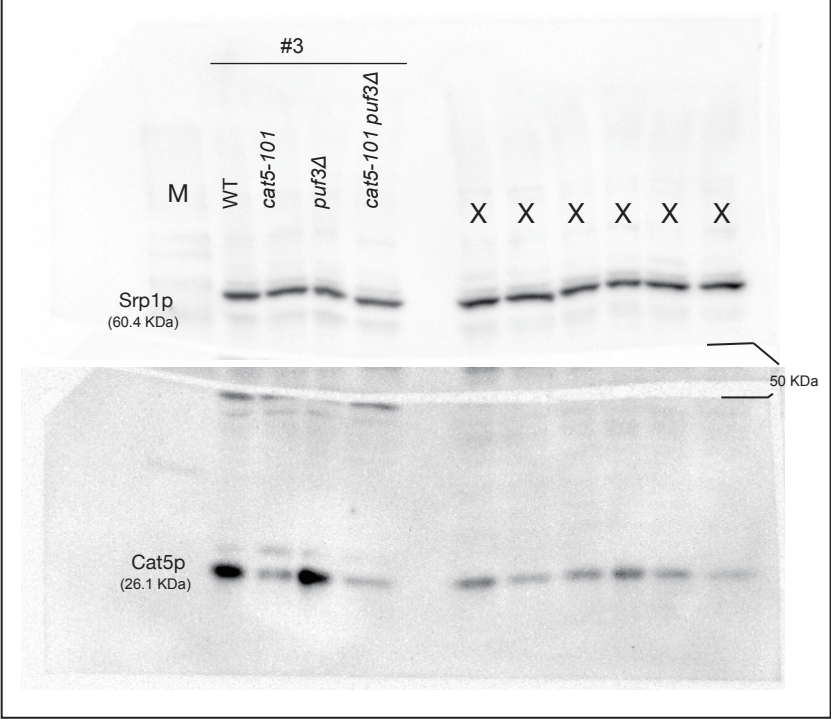

Fig. 3D

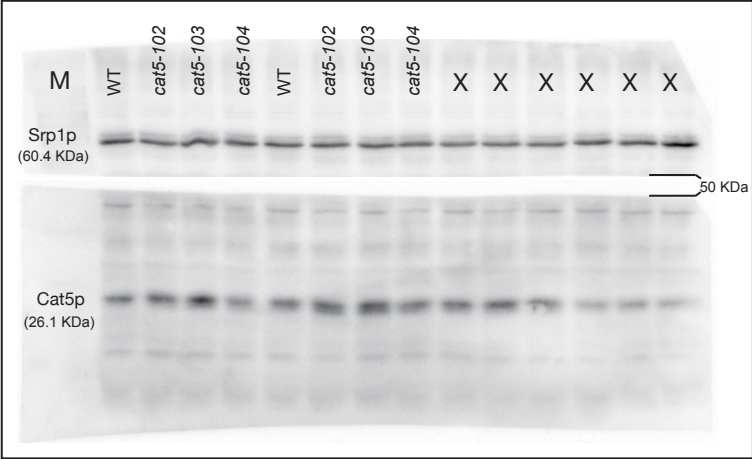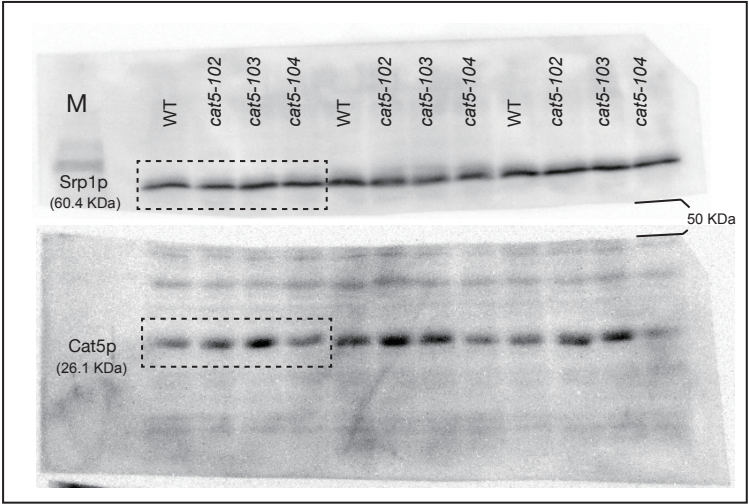

Fig. 3F

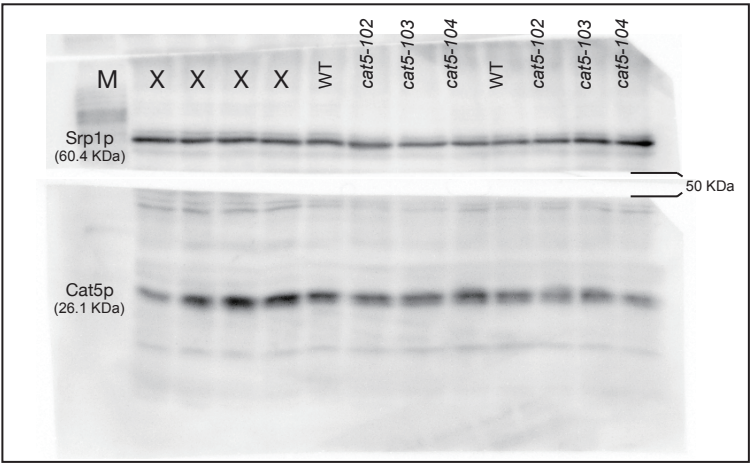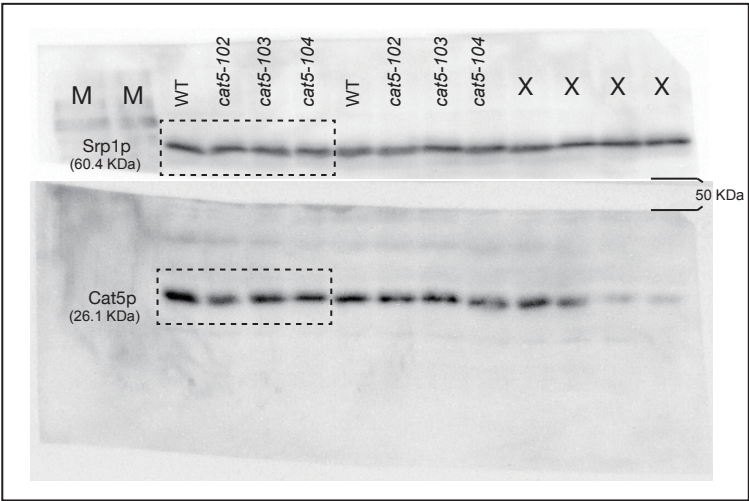

Supplement: S3 Raw images — (PDF) [file pone.0295659.s008.pdf]

Fig. 4A

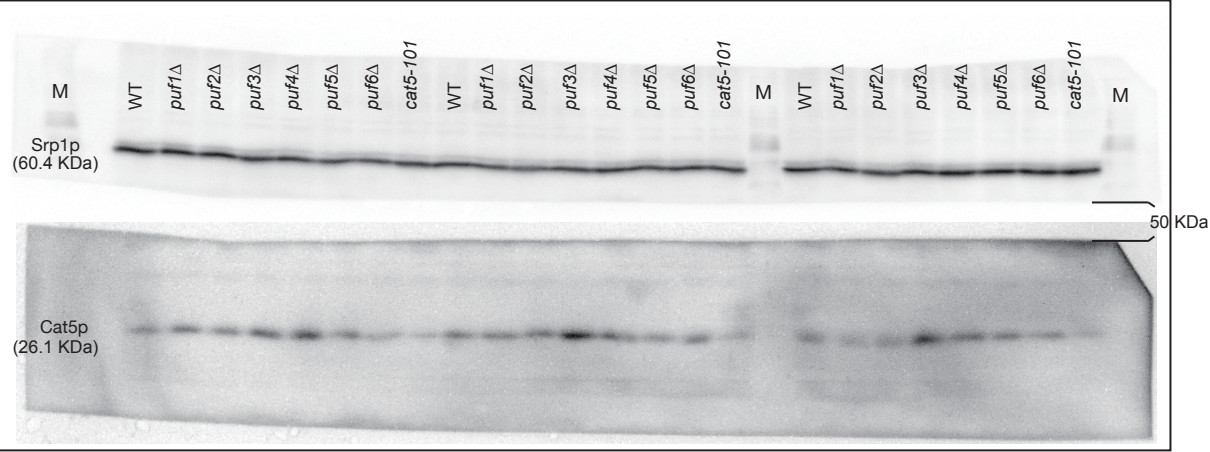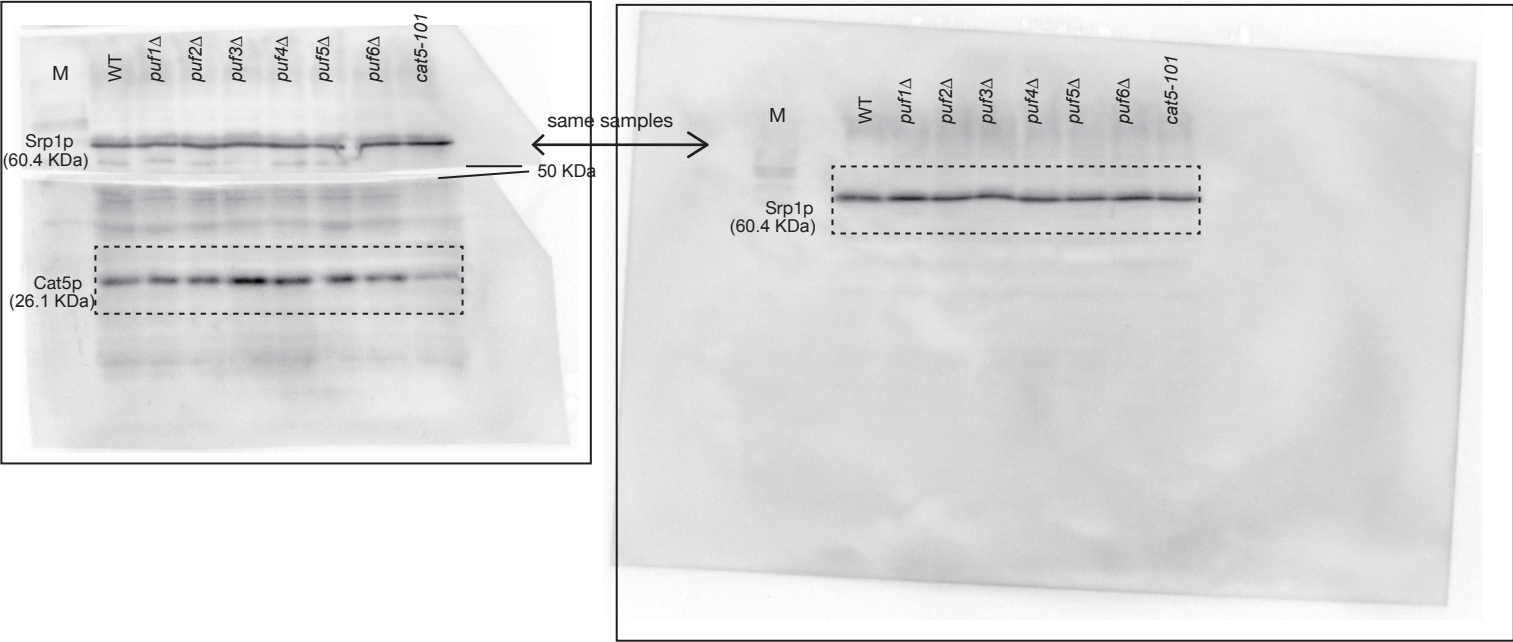

Fig. 4C

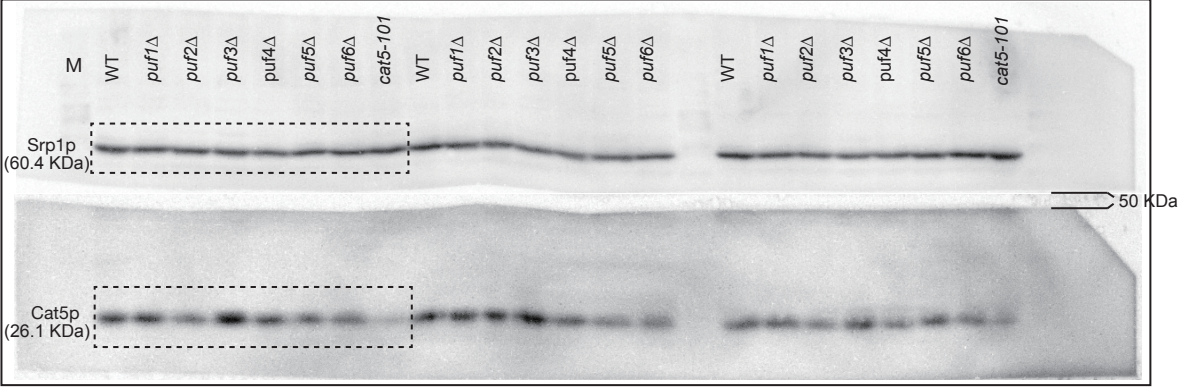

Supplement: S4 Raw images — (PDF) [file pone.0295659.s009.pdf]

Fig. 5A

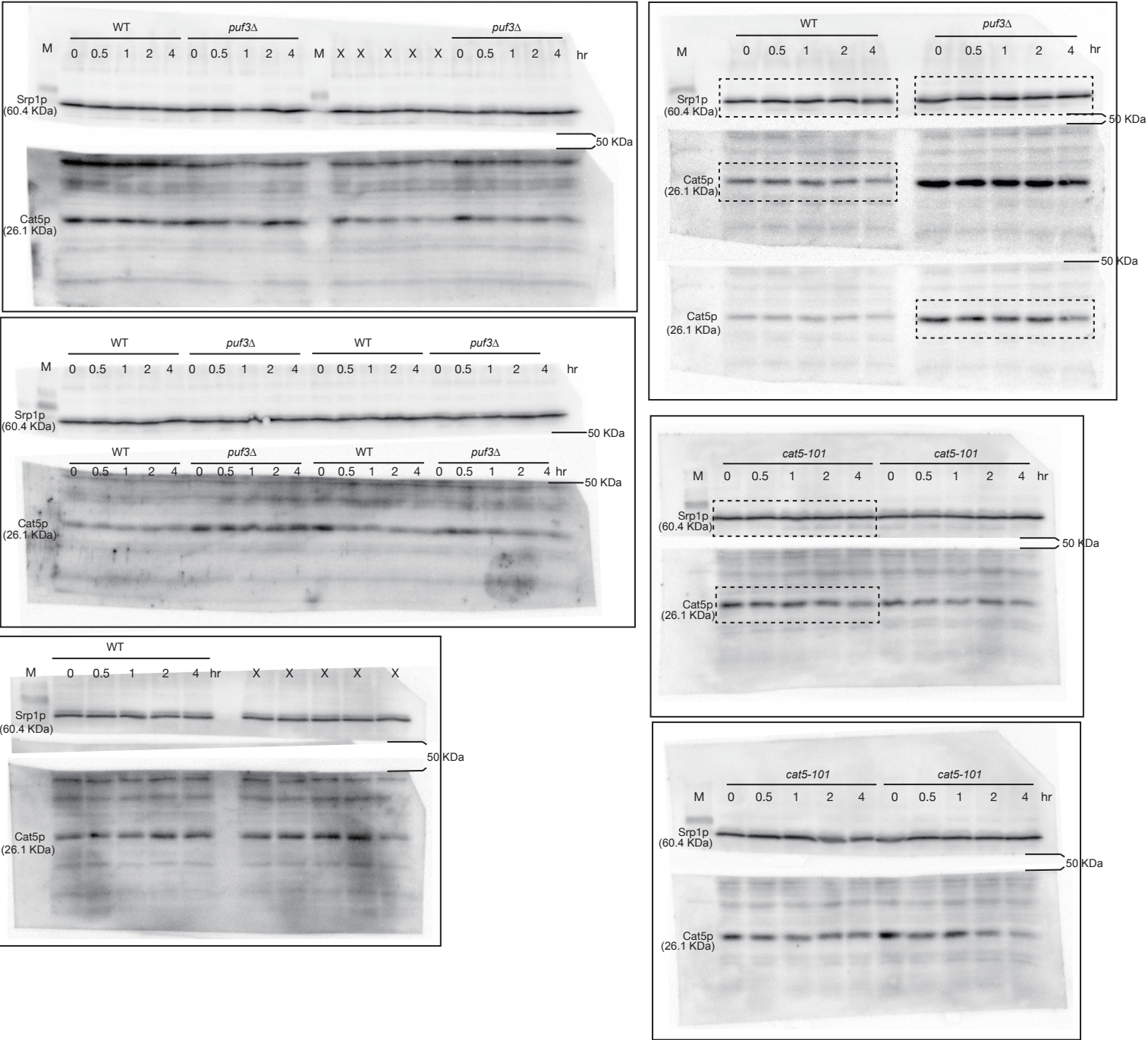

Fig. 5B

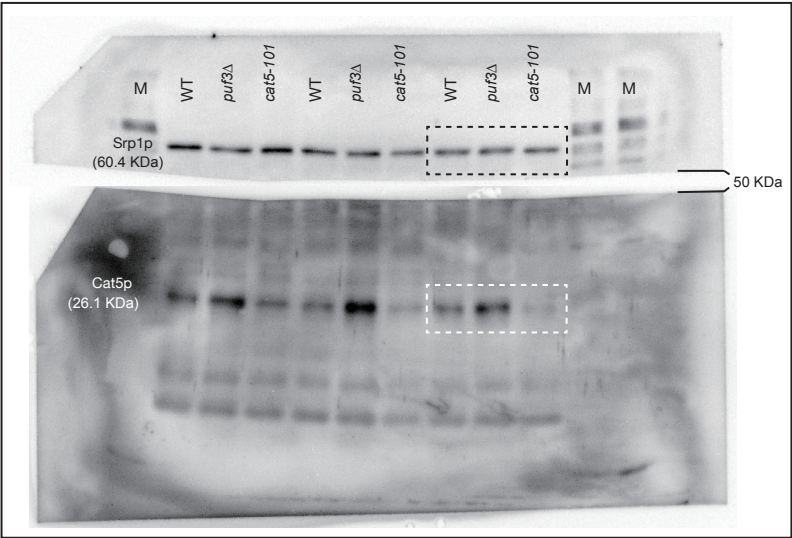

Fig. 5C

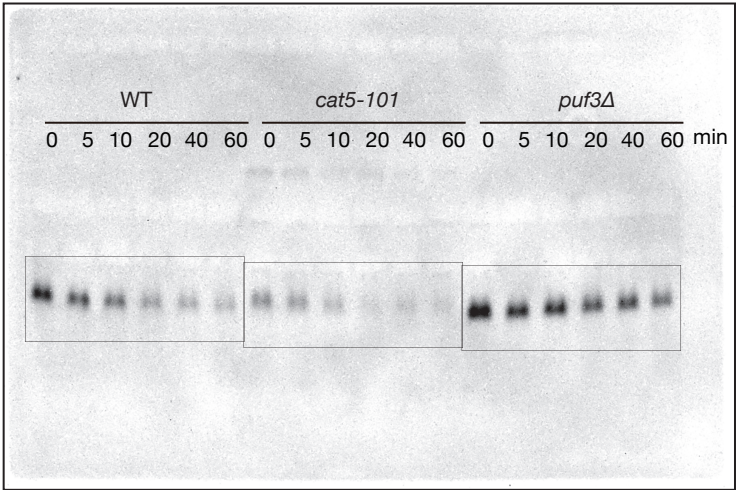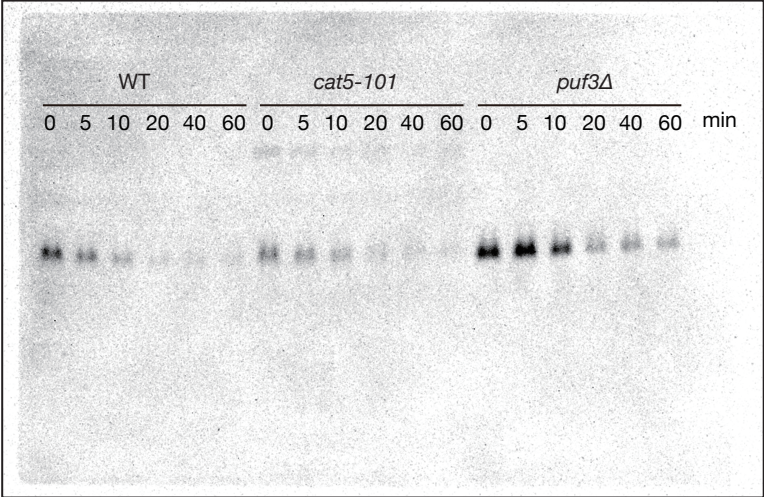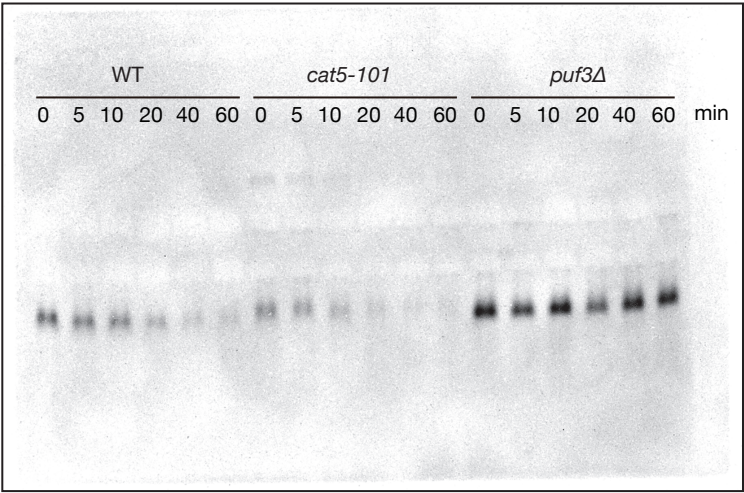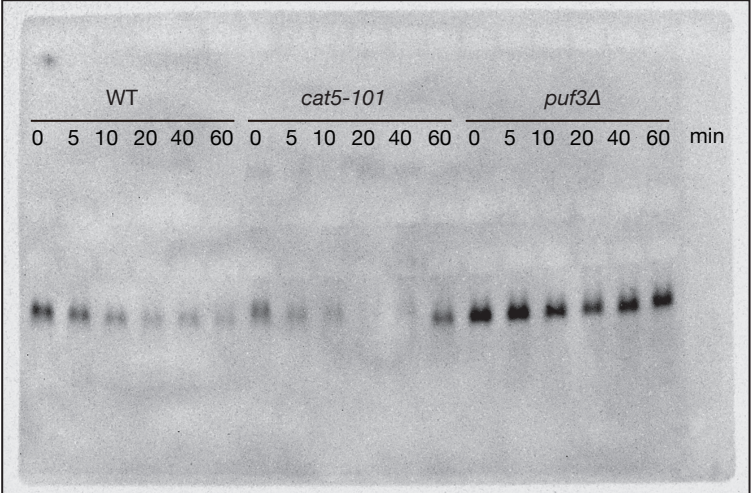

Supplement: S5 Raw images — (PDF) [file pone.0295659.s010.pdf]

Fig. S1A

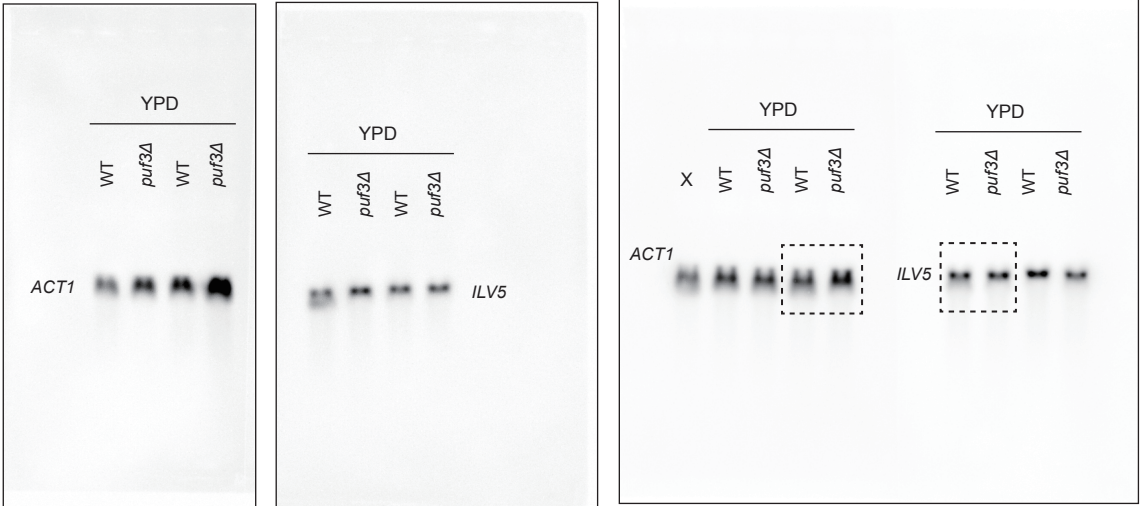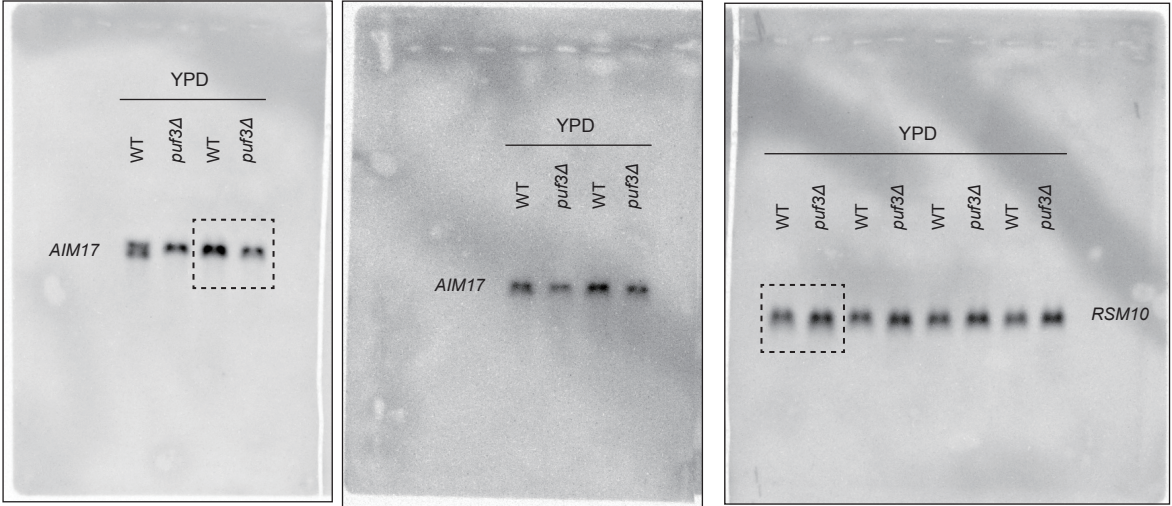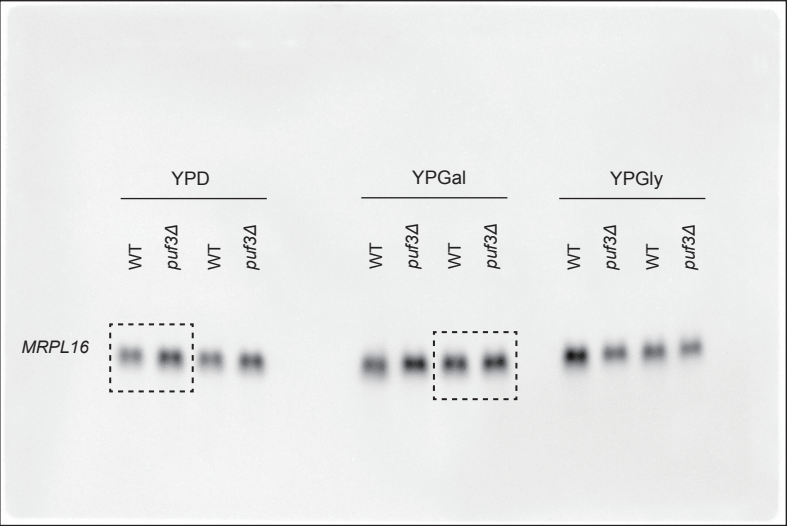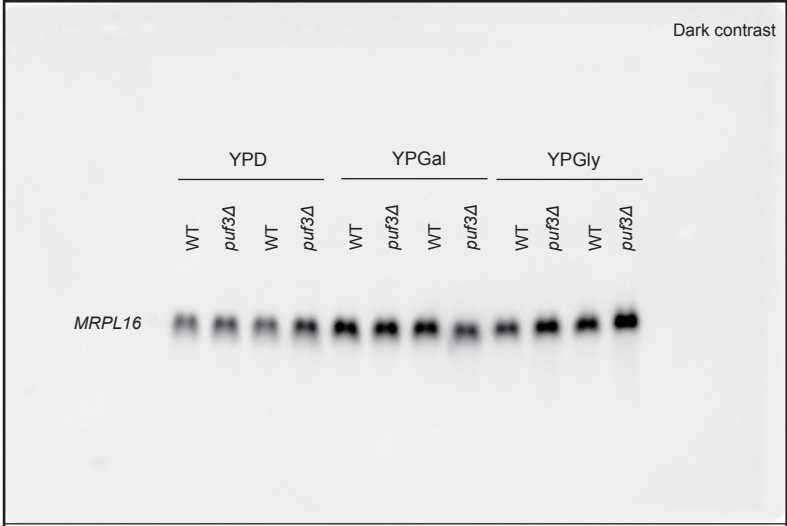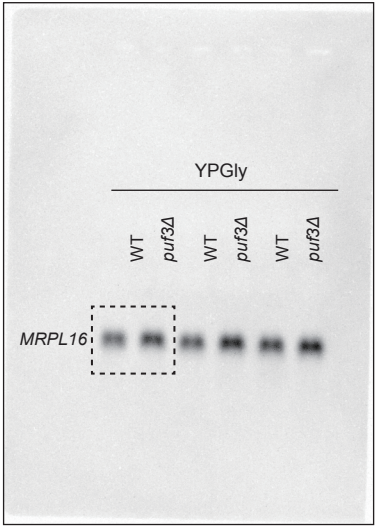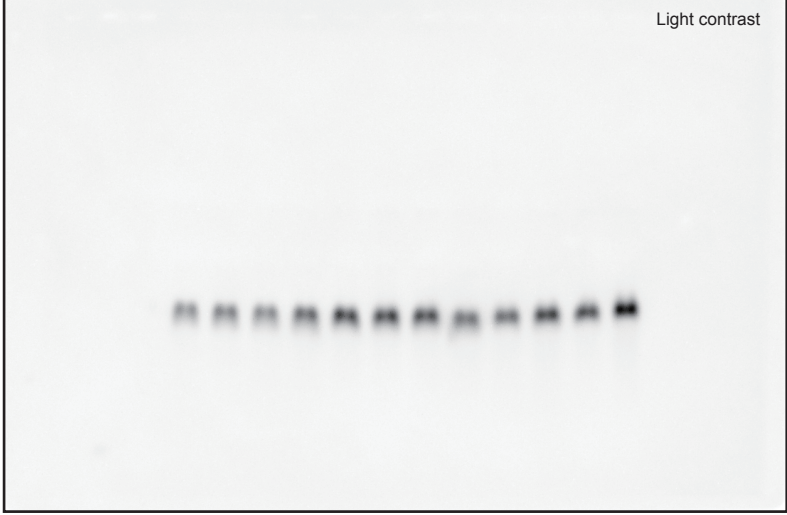

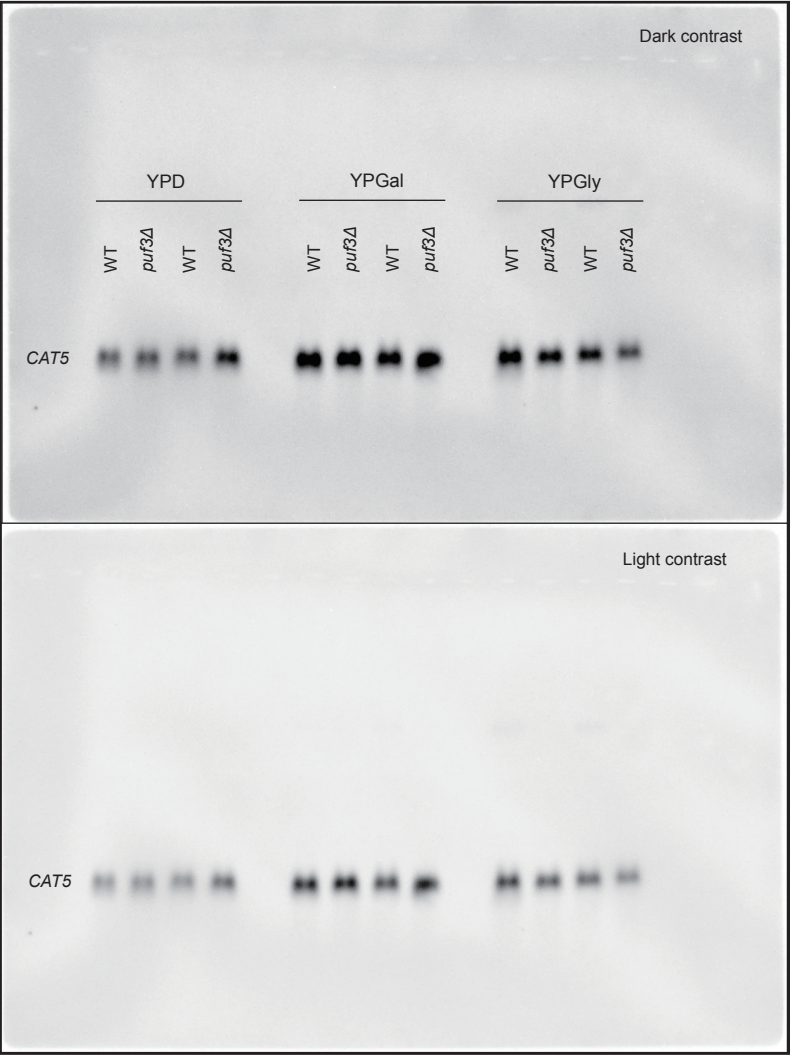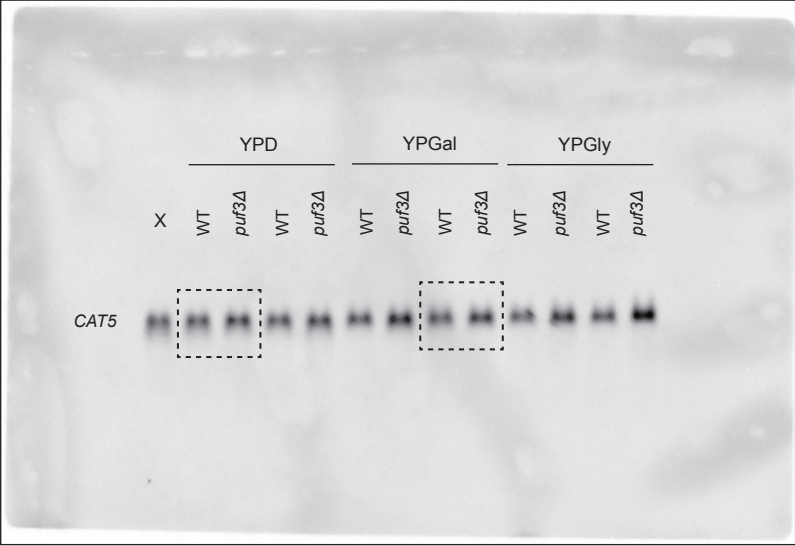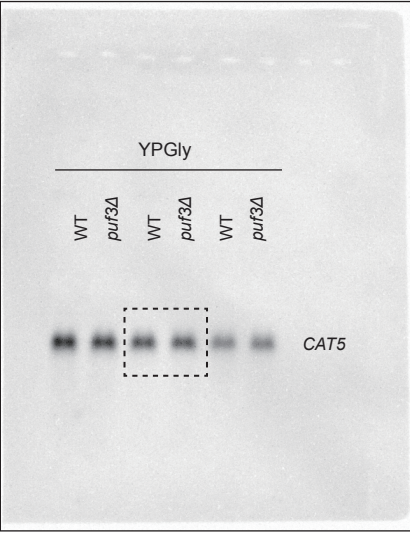

ACT1  
YPD

ILV5  
YPD

AIM17  
YPD

RSM10  
YPD

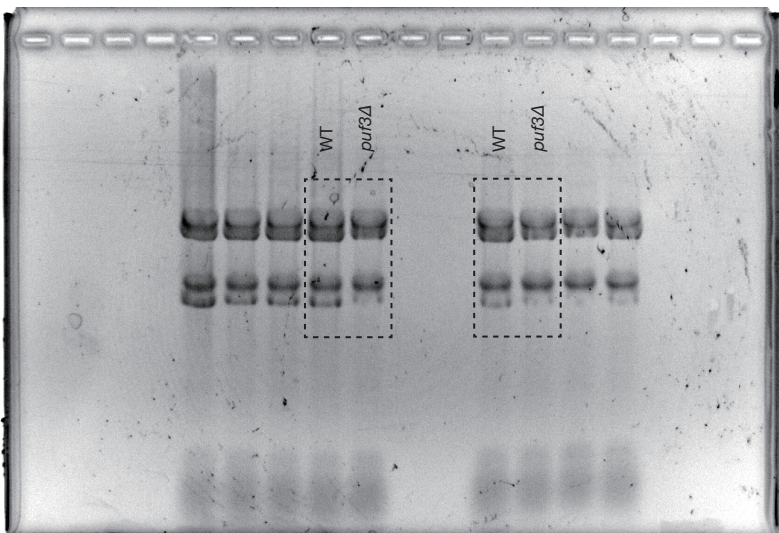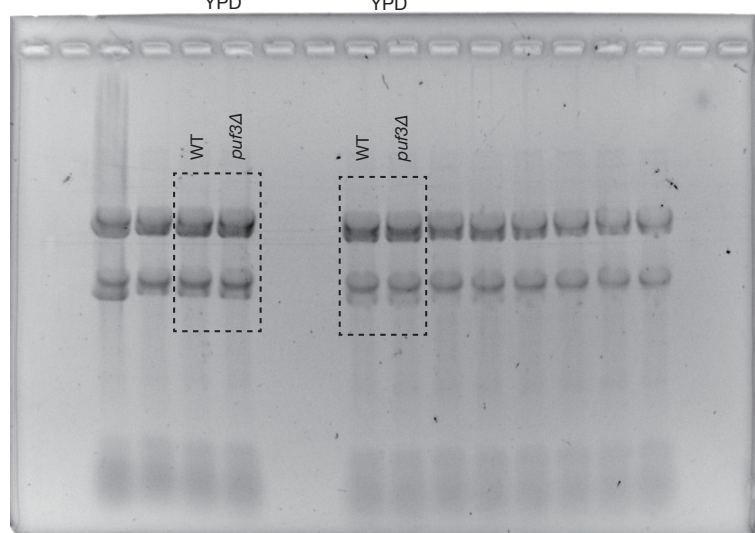

MRPL16  
YPD

MRPL16  
YPGal

CAT5  
YPD

CAT5  
YPGal

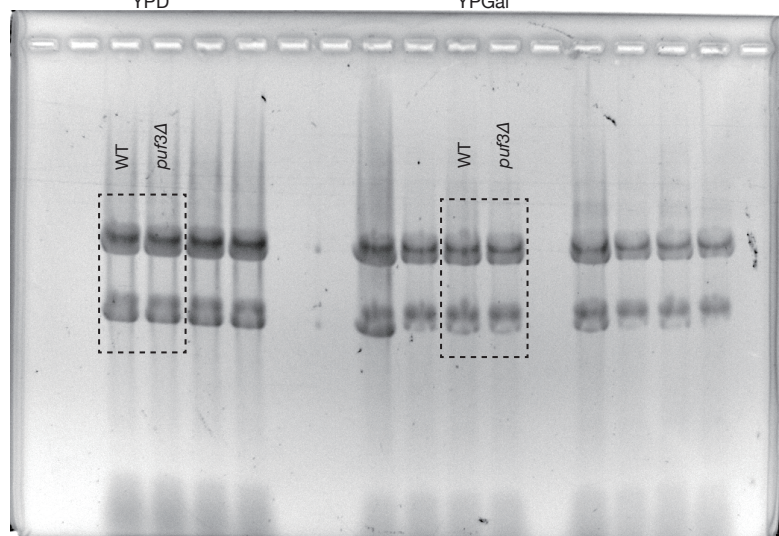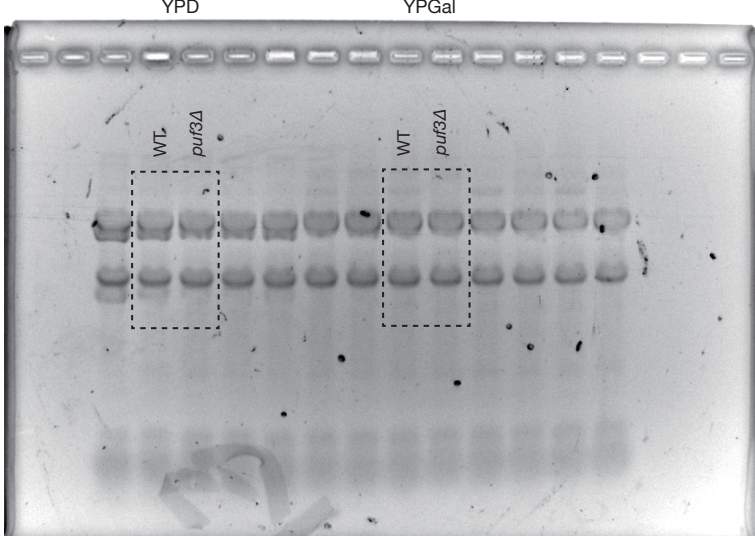

MRPL16  
YPGly

CAT5  
YPGly

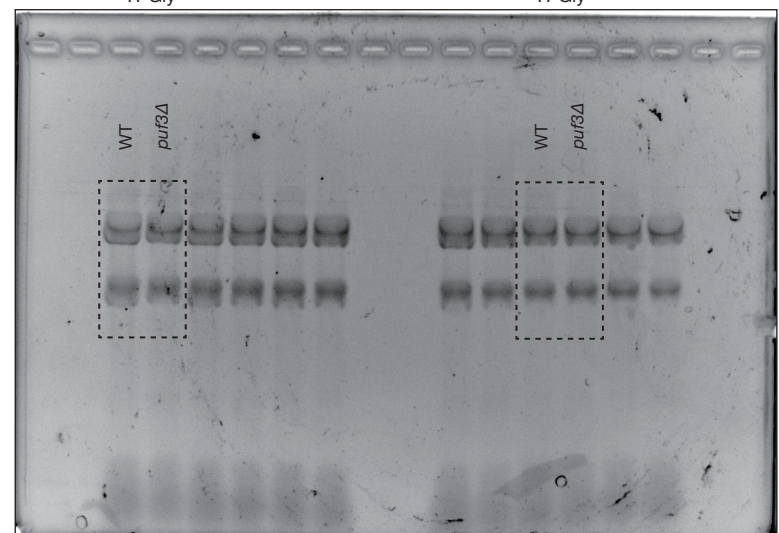

Supplement: S6 Raw images — (PDF) [file pone.0295659.s011.pdf]
